# Healthy Parent Carers programme: development and feasibility of a novel group-based health-promotion intervention

**DOI:** 10.1186/s12889-018-5168-4

**Published:** 2018-02-20

**Authors:** Aleksandra J. Borek, Bel McDonald, Mary Fredlund, Gretchen Bjornstad, Stuart Logan, Christopher Morris

**Affiliations:** 1 Peninsula Cerebra Research Unit (PenCRU), University of Exeter Medical School, St. Luke’s Campus, Heavitree Road, Exeter, EX1 2LU UK; 2 Peninsula Cerebra Research Unit (PenCRU) Family Faculty, University of Exeter Medical School, St. Luke’s Campus, Heavitree Road, Exeter, EX1 2LU UK

**Keywords:** Behaviour change, Intervention mapping, Wellbeing, Resilience, Empowerment, Peer support, Patient and public involvement, Disabled children, Parents, Carers

## Abstract

**Background:**

Parent carers of disabled children report poor physical health and mental wellbeing. They experience high levels of stress and barriers to engagement in health-related behaviours and with ‘standard’ preventive programmes (e.g. weight loss programmes). Interventions promoting strategies to improve health and wellbeing of parent carers are needed, tailored to their specific needs and circumstances.

**Methods:**

We developed a group-based health promotion intervention for parent carers by following six steps of the established Intervention Mapping approach. Parent carers co-created the intervention programme and were involved in all stages of the development and testing. We conducted a study of the intervention with a group of parent carers to examine the feasibility and acceptability. Standardised questionnaires were used to assess health and wellbeing pre and post-intervention and at 2 month follow up. Participants provided feedback after each session and took part in a focus group after the end of the programme.

**Results:**

The group-based Healthy Parent Carers programme was developed to improve health and wellbeing through engagement with eight achievable behaviours (CLANGERS – Connect, Learn, be Active, take Notice, Give, Eat well, Relax, Sleep), and by promoting empowerment and resilience. The manualised intervention was delivered by two peer facilitators to a group of seven parent carers. Feedback from participants and facilitators was strongly positive. The study was not powered or designed to test effectiveness but changes in measures of participants’ wellbeing and depression were in a positive direction both at the end of the intervention and 2 months later which suggest that there may be a potential to achieve benefit.

**Conclusions:**

The Healthy Parent Carers programme appears feasible and acceptable. It was valued by, and was perceived to have benefited participants. The results will underpin future refinement of the intervention and plans for evaluation.

## Background

Parent carers of disabled children are at increased risk of psychological and physical health problems. They commonly report higher levels of stress and depression [1–10] and poorer physical health [2, 3, 6, 7, 11] than parents of typically developing children. Evidence from Canada suggests parent carers’ health problems persist for many years and may worsen over time [12].

Multiple child, family and environmental factors can affect parent carers’ health and wellbeing, and might contribute to their poor health (see ‘needs assessment’ below). Some factors are difficult to change, but others could be more easily modified and therefore targeted by interventions. The particular life circumstances of parent carers may both have adverse effects on their health and be a barrier to participation in health promoting activities. These barriers may relate to difficulties with access because of the demands on their time and energy and to a feeling that activities may lack direct relevance to the complexities of their life experience. For this reason standard health promotion interventions may be inappropriate but there is a paucity of interventions that target parent carers’ and are specifically tailored for their needs.

The aims of this research were: (1) to develop an intervention to promote health and wellbeing of parent carers, and (2) to test the feasibility and acceptability of the intervention. In this paper, we describe the development of the Healthy Parent Carers intervention, and report on an initial testing of the feasibility of delivering the intervention.

## Methods

### Stakeholders involvement

The research had a strong ethos of meaningful engagement and partnership with parent carers as the intended ‘end users’ of the intervention. A group of parents of children with neurodisability from the research unit’s Family Faculty public involvement group worked closely with researchers to develop the intervention and the research plan. The working group met on 11 occasions and included 39 parent carers, of whom 21 attended at least one meeting during the development phase, and some contributed by phone or email.

Parent carers co-created the intervention by contributing to all stages of intervention development and testing. They proposed the idea for the project and helped to identify their specific needs through personal experiences. They advised on the design of the feasibility study, interpretation of its results, programme refinement, and future directions of this research. The details of the working group engagement, including meeting notes, are available online [13].

Other stakeholders consulted in this research included NHS health trainers, representatives from the local authority, and colleagues from the National Network of Parent Carer Forums and the Council for Disabled Children.

### Intervention development

The Healthy Parent Carers (HPC) programme was developed based on the Intervention Mapping approach [14], which is a systematic approach to developing health promotion interventions. We used six steps: (1) needs assessment, (2) developing programme outcomes and change objectives, (3) selecting methods and practical applications, (4) designing programme components, (5) testing intervention feasibility and acceptability and incorporating feedback, and (6) planning intervention adoption, implementation and evaluation. Table 1 outlines the key tasks completed in each step of the HPC intervention development (with Step 6 currently on-going).

**Table 1:** Steps and tasks undertaken in the intervention development

Steps in Intervention Mapping	Main tasks in the development of HPC intervention
Step 1: Needs assessment	1. Established a working group of parent carers in the PenCRU Family Faculty who were involved in the subsequent steps and tasks. 2. Conducted needs assessment through (i) consultations with parent carers, (ii) consultations with stakeholders, and (iii) literature search and review.
Step 2: Developing programme outcomes and change objectives	1. Specified and agreed on programme outcomes. 2. Specified and selected modifiable determinants of outcomes. 3. Determined change and performance objectives for programme outcomes (i.e. what needs to change and what steps need to be taken in order to meet programme outcomes), and created a matrix of change and performance objectives.
Step 3: Selecting methods and practical applications	1. Generated ideas for programme content and delivery with parent carers working group. 2. Identified and selected theory-based programme methods (i.e. behaviour change techniques) and practical applications to deliver intervention content and behaviour change techniques. 3. Specified intervention logic model.
Step 4: Designing programme components	1. Specified programme scope and themes, and designed programme materials, including the Facilitator Manual and the Guide for Parent Carers. 2. Reviewed programme materials with parent carers working group and pre-tested some of the activities includes in the programme. 3. Produced programme materials (i.e. Facilitator Manual, materials to be used in the group activities, Guide for Parent Carers), feedback and outcome assessment forms, and prepared tools to be used in the sessions.
Step 5: Testing intervention feasibility and acceptability and incorporating feedback	1. Planned a feasibility study to test feasibility of intervention delivery and acceptability of the intervention content and delivery; developed a study protocol; received ethics approval. 2. Recruited participants. 3. Delivered the group sessions; collected feedback from participants and facilitators. 4. Identified and incorporated the feedback and suggestions to improve the intervention design and delivery, as well as identified potential solutions to issues related to feasibility and implementation.
Step 6: Planning intervention adoption, implementation and future evaluation	1. Identifying potential stakeholders to involve in adoption and implementation of the intervention. 2. Reviewing and revising programme content, delivery methods and materials. 3. Specifying design of a pilot/evaluation study; developing an outcome and process evaluation plan. 4. Developing study protocol; piloting and evaluating the programme.

Based on Intervention Mapping approach [14]

#### Needs assessment (step 1)

In Step 1 we conducted a review of published research and consulted stakeholders to identify factors that affect parent carers’ health and wellbeing, and considered which of these could potentially be modified. We also sought to identify and appraise existing interventions for parent carers.

Some of the strongest predictors of mental health of mothers of disabled children identified in our needs assessment include participation in health-promoting behaviours, such as recreation, healthy diet and exercise, and time spent alone or on managing one’s health [15]. However, parent carers face specific challenges to engaging with health behaviours. These include constraints on their time and energy, insufficient breaks from their caring role or lack of qualified alternative caregivers [7]. Previous studies have also found that parent carers’ health was associated with their self-efficacy [1], feelings of guilt [5], locus of control and coping styles [16], self-esteem and self-mastery [17], and self-perceptions [18], all of which could potentially be targeted and modified by health-promoting interventions.

Many existing interventions for parent carers target external influences, for example by focusing on promoting parenting skills [19] or effectively navigating the healthcare services [20]. Other interventions target individual factors, but are limited in scope, for example by focusing on treating stress [19] or providing emotional support [21] rather than actively promoting health and wellbeing. A systematic review of psychological therapies for parents of children with chronic illness suggested promising results in terms of improved parent mental health [22]. Problem solving therapy was found to be effective for improving parent mental health. No benefits were found for cognitive behavioural therapy or family therapy on parent outcomes. However, the quality of the evidence was low and few relevant trials were found. A systematic review of mindfulness interventions for parents of children with autism indicated potentially positive effects on parents’ stress and psychological wellbeing with studies reporting good attendance and retention in 8-week programmes [23]. We found no interventions targeting important factors identified by our Working Group in the existing literature; that is interventions targeting both physical health and mental wellbeing, focused on parent carers’ outcomes, and involving a range of behaviours that can be tailored to parents’ needs, preferences and opportunities.

#### Programme outcomes and performance objectives (step 2)

Step 2 involved specifying (i) who and/or what will change as a result of the intervention (programme outcomes), (ii) what participants will need to do to achieve these outcomes (performance objectives), and (iii) factors associated with performance of behaviours (determinants of change).

In terms of behavioural outcomes, our parent carers’ working group recommended that the programme promotes engagement with a wide range of small, everyday behaviours from which parents could choose, depending on their specific circumstances and needs. They suggested that this would be more empowering, acceptable and feasible to parent carers than promoting specific behaviours (e.g. a healthy diet). A set of health-promoting behaviours linked with health and wellbeing was identified. These behaviours have been promoted as evidence-based ‘Five Ways to Wellbeing’ [24, 25] and ‘CLANGERS’ [26]. They include: (1) Connecting with people, (2) continuing Learning, (3) being Active, (4) taking Notice (or being mindful), (5) Giving, (6) Eating well, (7) Relaxing, and (8) maintaining Sleep hygiene. These behavioural targets were discussed with parent carers and perceived to be potentially more difficult for parent carers. Hence, in order to make this generic public health advice specific to parent carers’ circumstances, it was tailored by including parent carer-specific examples of behaviours, barriers to health behaviours, and problem solving.

In addition, we identified psychological outcomes, such as increasing a sense of empowerment and resilience, necessary to engage with changing health-related behaviours. Empowerment is a sense of agency or internal locus of control, whereas resilience is an ability to cope with adversities, problems and barriers. Both are likely to be particularly important to parent carers who often face, and need to cope with, many factors related to their care-taking role (as discussed in our Working Group).

Programme outcomes were broken down into smaller, observable actions (i.e. performance objectives). As we had a number of outcomes, and given that parents’ baseline levels and approaches to achieving them will vary, we formulated generic actions that can be taken to achieve them (i.e. ‘steps to making lifestyle changes’) (Table 2).

**Table 2:** Performance objectives, determinants of change, theoretical methods and practical applications in the HPC programme

Performance Objectives (i.e. actions to achieve intervention outcomes)	Determinant of Change	Selected Theoretical Methods (Behaviour Change Techniques, BCTs)	Practical Applications for BCTs (Delivery strategies)
1. Understand the behaviours (i.e. CLANGERS) and psychological outcomes (i.e. resilience, empowerment)	1. Knowledge (i.e. understanding of the links between health and behaviours, awareness of risks and consequences, awareness of current behaviours)	1. Provide/exchange information about behaviour-health link	Booklet, group brainstorming and discussions
2. Understand the link with health and wellbeing	2. Attitudes (i.e. beliefs about consequences of action and inaction)	2. Provide/exchange instructions on practical strategies	Booklet, group brainstorming and discussions
3. Assess your current situation (i.e. self-assessments, self-monitoring)	3. Self-efficacy (i.e. belief about capabilities, confidence in one’s ability)	3. Prompt barrier identification and problem solving	Booklet, group brainstorming and discussions
4. Build motivation for change (i.e. identify personal reasons for change)	4. Social support (i.e. emotional and practical social support within and outside the group)	4. Prompt practice	Practical group activities
5. Identify practical strategies	5. Skills (i.e. learning and practice of skills for behaviour change skills and for health behaviours)	5. Prompt specific goal setting	Booklet, worksheets used in the sessions
6. Set specific goals (action plan)		6. Prompt use of prompts and rewards	Booklet
7. Perform the behaviours		7. Prompt self-monitoring	Booklet, worksheets to use between sessions
8. Re-asses your situation (i.e. self-monitor, review, reflect)		8. Prompt goal/progress review	Booklet, group discussions
9. Revise or adjust the behaviour, goal/action plan		9. Provide general support and encouragement	Group discussions and group activities
		10. Provide opportunities for social comparisons	Group activities

Subsequently, we selected determinants of change. As the evidence on determinants specific to parent carers is limited, we selected constructs from the Theoretical Domains Framework [27, 28] based on general evidence of associations with behaviour change, needs assessment and consultation with the working group. These included knowledge, attitudes, self-efficacy, social support, and skills (i.e. skills for behaviour change, such as goal setting or problem solving, and for performance of behaviours, such as relaxation techniques).

#### Theoretical methods and practical applications (step 3)

**Fig. 1:**
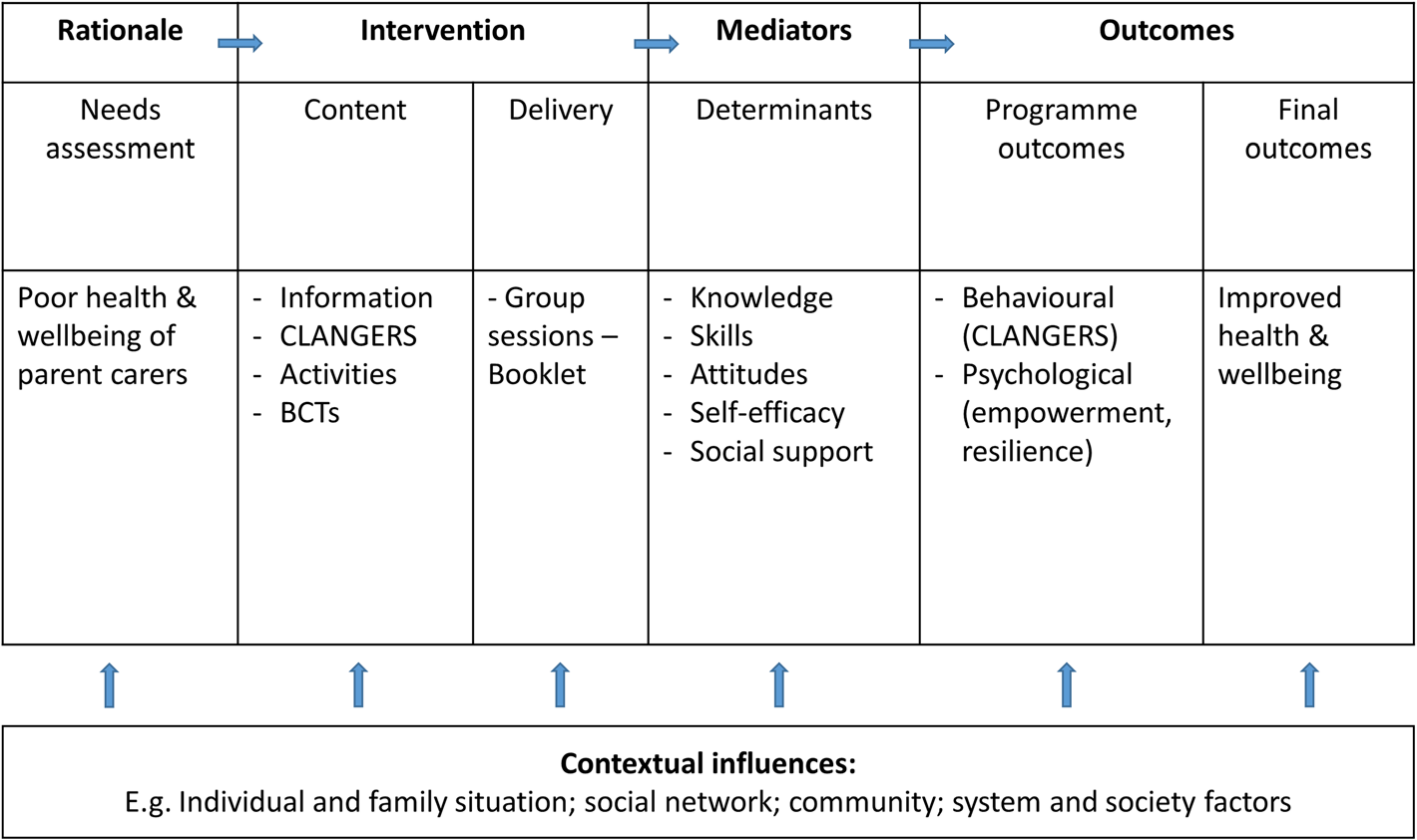
Logic model of the Healthy Parent Carers intervention

In Step 3, drawing on a taxonomy of behaviour change techniques (BCTs) [29] and evidence showing associations of BCTs with effectiveness of health interventions [29–32], we selected evidence-based methods that were relevant to the outcomes and objectives of the HPC intervention. Through consultations with the working group, we selected modes of delivery and practical strategies to deliver intervention content with BCTs (Table 2). Two modes of delivery were selected: a printed participant booklet (the Guide for Parent Carers) and group sessions. An intervention logic model was also developed (Fig. 1).

#### Programme components (step 4)

In Step 4, we designed and produced programme materials for participants and facilitators. The Guide for Parent Carers was intended to be used between the sessions and after the programme ended. It included the same topics as covered in the group sessions divided into three parts: (1) understanding health and wellbeing (i.e. factors affecting health and wellbeing, health-promoting behaviours, resilience and empowerment, self-assessment); (2) taking steps to better health and wellbeing (i.e. CLANGERS, goal-setting and self-monitoring worksheets); and (3) planning for the long-term (i.e. building resilience and managing stress, self-assessment and reflecting on progress, setting long-term maintenance goals). In addition, we created a website for parent carers with additional resources relevant to the HPCs programme.

A Facilitator Manual included detailed session outlines, instructions and timings for the activities, and materials to be used in group activities, such as URLs for videos and worksheets. The intervention was designed to be delivered sequentially following the Facilitator Manual, but some degree of flexibility within the sessions was possible.

The Guide for Parent Carers and activities included in the Facilitator Manual were discussed, pre-tested and refined with the parent carers in the working group. In addition, key recommendations on planning and reporting health interventions, education and training were consulted; these included the NICE Guideline on Behaviour Change [33], the Template for Intervention Description and Replication (TIDieR) [34], and the checklist for group-based behaviour-change interventions [35].

### Feasibility study (step 5)

We conducted a feasibility study to test (i) the feasibility of delivery of the HPC programme and (ii) its acceptability to participants and peer facilitators (Intervention Mapping Step 5). Specifically, the study aimed to assess: strategies for recruitment and selection of participants, delivery of the programme and facilitation of group sessions, intervention content, participants’ session attendance and retention, and participants’ and facilitators’ programme feedback.

The University of Exeter Medical School ethics committee approved the study (REC 15/11/084) and all participants documented their informed consent to participate.

#### Feasibility study methods

The study was advertised online on the research group’s website and social media of relevant local organisations for parent carers. Participants were also recruited through personal networks of parent carers involved in the working group. The recruitment was conducted between December 2015 and January 2016, and the six group sessions were delivered between the end of January and beginning of March 2016. We sought to recruit a minimum of six participants to constitute ‘a group’.

Potential participants expressed interest by contacting the research unit. A researcher explained the study and conducted a preliminary screening by phone. Participants who could not attend six sessions were offered a one-off introductory session. A researcher and the group facilitator then met each potential participant to provide a more detailed explanation of the study, answer any questions, and to screen for inclusion.

Inclusion criteria included self-identification as a primary carer of a child or young person with additional needs and/or disabilities under 25 years (consistent with current UK Department of Health and Department of Education Special Educational Needs & Disability (SEND) legislation and The Children’s Act). Potential participants had to be willing and able to attend the sessions on pre-scheduled dates, be able to communicate in English, not participated in the intervention development, and had no symptoms of severe depression or suicidal ideation identified using the Patient Health Questionnaire (PHQ-9) [36, 37]. A risk protocol was in place if any concern arose at the screening or during sessions. Volunteers who met the inclusion criteria were invited to participate in the study.

#### Intervention

The HPC programme was delivered in a small group setting. We aimed to include between 6 and 15 participants in the group; the actual group included seven participants. The two female peer facilitators who delivered the programme were involved in the development of the HPC programme from inception and co-designed the Facilitator Manual. They were also experienced in delivering training to parent carers and facilitating parent carers’ groups. Due to their involvement in the programme development and relevant experience, no further training was seen as necessary, but on-going support and supervision were provided. The group sessions took place in a university seminar room, with tables arranged in a horseshoe shape facing the facilitators, a screen to view online videos, and a whiteboard on which discussions were noted in the form of mind-maps, photographed, and sent to participants.

The facilitators delivered the sessions following the Facilitator Manual. There were 6 weekly 3-h group sessions, with 1 week break in the middle due to school holidays. Each session was structured in a similar way: starting with an introduction and ice-breaker, review of the week, introducing each topic through group brainstorming, followed by one or two activities to illustrate the topic, individual action planning, recap of the session and conclusion. Before each session there was an additional half an hour for arriving, tea and coffee and informal conversations, and at the end of each session there was another half an hour for lunch and more informal interaction. Beverages and lunch were provided at sessions and participants were offered reimbursement of travel costs; they were not paid for participating in the programme or offered other inducements. Session topics and exemplary activities are in Table 3. The sessions were interactive, based around group discussions and sub-group activities. Although the sessions and main discussions were structured and outlined in the Facilitators Manual, a degree of flexibility for tailoring group discussions was possible.

**Table 3:** Healthy Parent Carers programme: outline of session content and activities

Session	Topics and group activities
Session 1: Introduction to health and wellbeing	• Introductions and agreeing group ground rules • Health and wellbeing and what influences them for parent carers (group discussion) • Introduction to CLANGERS • Resilience (group game and discussion) • Managing responsibilities (group activity and discussion) • Self-assessment of health and wellbeing • Action planning
Session 2: Connect and Learn	• Ice-breaker activity, review of the week (group sharing) • Introducing ‘Connect’ (group brainstorming) • Personal connections and sources of support (group activities and discussions) • Introducing ‘Learn’ (group brainstorming) • Learning to make a paper box (group activity) • Action planning
Session 3: Be Active and Notice	• Ice-breaker activity, review of the week (group sharing) • Introducing ‘being Active’ (group brainstorming, video, group discussion) • Introducing ‘taking Notice’ (group brainstorming, video, group activities and discussion) • Action planning
Session 4: Give and Eat Well	• Ice-breaker activity, review of the week (group sharing) • Introducing ‘Give’ (group brainstorming, activities, and group discussion) • Introducing ‘Eat well’ (group brainstorming, video, group activity and group discussion) • Action planning
Session 5: Relax and Sleep	• Ice-breaker activity, review of the week (group sharing) • Introducing ‘Relax’ (group brainstorming, activities, and group discussion) • Introducing ‘Sleep’ (group brainstorming, activity, and group discussion) • Action planning
Session 6: Keeping healthy	• Ice-breaker activity, review of the week (group sharing) • Managing stress (group brainstorming, video, activity and group discussion) • A group walk • Reflecting on progress, self-assessments of health and wellbeing • Long-term action planning • Recap of the programme and conclusions

#### Measures

The main outcomes were feasibility of delivery and acceptability of the intervention to participants and facilitators, with pre-defined criteria for judging success (as listed in Table 5). To assess feasibility we collected information on recruitment (number of interested and eligible participants, recruitment channels), attendance (including reasons for missing any sessions) and retention in the programme. Acceptability was assessed through participants’ feedback using questionnaires at the end of each session. We used rating scales (scored 1–5 where 1 indicated least and 5 most satisfied) to assess satisfaction with delivery, content, relevance, perceived helpfulness and likely impact (i.e. whether participants intended to make any changes in result of the session and if yes, what would these be). We also collected free-text comments on favoured elements and/or suggestions for improvements. A week after the final session, the researchers conducted an audio-recorded focus group with the participants. Feedback from facilitators was collected through de-briefing meetings with researchers at the end of each session. Fidelity of session delivery was assessed in de-brief meetings and session audio recordings.

Additionally, we collected quantitative data on intervention outcomes in order to test assessment methods. We assessed ‘health utility’ using the EuroQol 5 Dimensions questionnaire (EQ-5D) [38], depression symptoms with the PHQ-9 [36, 37], and wellbeing with the Warwick-Edinburgh Well-Being Scale (WEMWBS) [39]. Measures were taken on three occasions: before the intervention, at the end of the programme and 2 months after the programme was completed.

The EQ-5D is recommended by NICE and is commonly used in health economic evaluations to measure health utility. The version used had five questions, each with three response options. Health utility scores are calculated from self-reported health states and weighted according to preferences for health states from a UK reference population [40]. The PHQ-9 questionnaire is recommended by NICE to assess depression in adults [39], and its use is highlighted in NHS clinical pathways [41, 42]. It has nine items with four response options; individual responses are scored 0 to 3 and then summed to produce a score from 0 to 27. Scores of 20 and above are considered indicative of severe depressive symptoms [43]. The WEMWBS was developed and validated as a measure of mental wellbeing in general populations and to evaluate interventions that aim to improve mental wellbeing [39]. It has a 14-item scale with five response categories, summed to provide a single score ranging from 14 to 70. The items measure both emotional and functional aspects of mental wellbeing.

#### Analysis

Quantitative data from feedback forms and questionnaires were analysed using descriptive statistics. Qualitative data including comments from feedback forms, de-brief meetings and the focus group were analysed thematically by identifying opinions about the programme and the sessions, perceived impact, and suggestions for improvements.

## Results

### Feasibility

We received 12 expressions of interest in participation. Telephone screening identified that one person had been part of the working group, two could not commit to attending the six sessions, one was not able to participate and one could not be contacted.

### Participant characteristics

Seven parent carers, all white British women, met the inclusion criteria and signed up to participate in the programme (Table 4). The participants and their children were broadly of a similar age; the children’s conditions were a mix of physical and intellectual disability. They lived in diverse circumstances, some in a city and others in villages. The Indices of Multiple Deprivation [44] in the areas where the participants lived were mixed; four lived in areas that are relatively more deprived relative to England as a whole.

**Table 4:** Characteristics of participants in the Healthy Parent Carers feasibility study

Participant age		Mean 44.6 (range 37–53) years
Partner at home		6 yes, 1 no
Ethnicity		100% white British
Child diagnosis	Cerebral palsy	2
	Autism/ADHD	4
	Undiagnosed	1
Child age		Mean 9.8 (range 6–13) years
Siblings		Median 1 sibling, range 0–4
Indices of Multiple Deprivation^a^ in area of residence (by postcode): national quintiles	1 (least deprived)	1
	2	1
	3	1
	4	4
	5 (most deprived)	0

^a^The Indices of Deprivation 2015 [42] provide a set of relative measures of deprivation for small areas across England, based on seven different domains of deprivation: (i) Income Deprivation, (ii) Employment Deprivation, (iii) Education, Skills and Training Deprivation, (iv) Health Deprivation and Disability, (v) Crime, (vi) Barriers to Housing and Services, and (vii) Living Environment Deprivation. Each of these domains is based on a basket of indicators

### Engagement

Three participants each missed one of the sessions due to prior commitments, which were known in advance; each session was attended by at least five participants. All seven participants remained involved in the study throughout the programme (i.e. there was no attrition); six parents attended a focus group and completed follow-up questionnaires, and six participants and the two facilitators attended an informal, social catch-up meeting approximately 3 months after the end of the intervention that was requested by several participants when the programme and study had finished.

### Fidelity of delivery

Group sessions were delivered with fidelity accordingly with the Facilitator Manual, which was assessed by the researchers (AB and CM) through discussion at the de-brief meetings with facilitators (comparing delivery with session plans). Few modifications were made to the Facilitator Manual based on the facilitators’ feedback provided in de-brief meetings (e.g., adapting some group activities) but these were made prior to the sessions and subsequently delivered as planned.

Overall, in comparison to our pre-defined criteria for judging the study as feasible, we had successful results in terms of attendance and retention, and the only aspect that did not meet our criteria was recruitment (Table 5).

**Table 5:** Comparison of the results with criteria for study success

	Criteria for study success	Results
Feasibility		
Recruitment	Recruiting at least 20 participants within a month.	12 expressions of interest and seven participants included.
Attendance	Majority of participants attending at least 5 out of 6 sessions.	All participants attended at least 5 out of 6 sessions.
Retention	Retaining at least 70% of participants at the 2-month follow up.	No participants dropped out from the programme. All participants remained in the programme until Session 6, and all returned the post-intervention and follow-up questionnaires.
Acceptability		
		(mean, % scores 4-5^a^)
Satisfaction	High participants’ overall satisfaction with the programme (≥80% scores 4–5).	4.2 (83%)
Delivery	High participants’ satisfaction with the programme delivery (≥80% scores 4–5).	4.3 (94%)
Content	High participants’ satisfaction with the programme content (≥80% scores 4–5).	4.7 (100%)
Relevance	High participants’ perception of relevance of the programme (≥80% scores 4–5).	4.4 (98%)

^a^Mean score on 1 to 5 scale, where score 4 indicated ‘satisfied’ and 5 ‘very satisfied’

### Acceptability

All participants completed feedback forms at the end of each session they attended. Overall, the results met our pre-set criteria for judging the programme as acceptable (Table 5). At least 80% of responding participants were ‘satisfied’ or ‘very satisfied’ (i.e. scoring 4 or 5) with each session (Table 6). At the end of the programme, five out of six responding participants were satisfied with the programme and would recommend it to other parent carers.

**Table 6:** Participants’ session feedback: quantitative results

N of participants^a^	Session 1 *n* = 6	Session 2 *n* = 6	Session 3 *n* = 6	Session 4 *n* = 5	Session 5 *n* = 6	Session 6 *n* = 7
Overall, how satisfied are you with the session?^b^	4.3 (83%)	4.3 (100%)	4.5 (83%)	4.2 (80%)	4.2 (100%)	4.1 (86%)
How satisfied are you with the session’s:						
Content^b^	4.2 (100%)	4.5 (100%)	4.5 (100%)	4.2 (80%)	4.2 (100%)	4.1 (86%)
Delivery^b^	4.7 (100%)	4.7 (100%)	4.7 (100%)	4.8 (100%)	4.8 (100%)	4.6 (100%)
Relevance^b^	4.3 (100%)	4.5 (100%)	4.5 (100%)	4.4 (100%)	4.5 (100%)	4.3 (86%)
Do you feel that you were able to take active part in the session?^b^	5.0 (100%)	4.7 (100%)	4.7 (100%)	4.2 (80%)	4.3 (83%)	4.6 (86%)
How useful was the session in helping you to improve your health & wellbeing?	3.5 (50% useful)	4.2 (100% useful)	4.3 (100% useful)	4.0 (75% useful)	3.7 (50% useful)	4.0 (71% useful)
Will you make any changes as a result of attending the session/programme?	100% yes	100% yes	83% yes	80% yes	50% yes	86% yes

^a^In total the group comprised seven participants, but three participants missed one session each and not all attending participants completed feedback forms (which were optional) after each session, resulting in missing data

^b^Mean score on 1–5 scale, where score 4 indicated ‘satisfied’ and 5 ‘very satisfied’ (% respondents scoring 4 or 5)

Responses to open questions in the feedback forms indicated that the participants valued the programme for the group context (i.e. meeting and identifying with other parent carers, thus providing opportunities for sharing experiences and peer support in a positive group setting) and learning from the programme and others (i.e. becoming aware of doing CLANGERS, giving oneself permission to take care of, and prioritise, own health and wellbeing) (Table 7).

**Table 7:** Participants’ programme feedback: qualitative results

Themes	Illustrating quotes
Satisfaction with the programme and its impact	
Overall satisfaction	• ‘It was really good… I learnt a lot. I’ve never heard of CLANGERS, so that was new… And obviously meeting new people was great, and [the facilitator] made it fun, and it was really informative.’ (FG) • ‘I found all of that absolutely brilliant and it’s made me think so much more. I really really really enjoyed the course.’ (FG) • There were times in my life when this [course] would have been a life saver. Coming somewhere like this would have saved my life. It would have made such a difference to me being good and not very good.’ (FG)
Impact and changes post-programme	• ‘On Friday I parked further away from school… just because in my head I’m thinking it’s better for my children to walk a little bit, and I wouldn’t have done that if hadn’t come here.’ (FG) • ‘I walked down to meet [another participant], whereas I would’ve just driven. It’s only 10 min walking but I would’ve always just driven down.’ (FG) • ‘I’m forever going up and down the stairs, but normally I just plot up, so I’ve started to run up them more cause I’m thinking ‘I might not be going for that 30 min walk but the amount of time, especially when kids are at home, I’m running up and down the stairs…” (FG) • ‘The Eat Well – I’ve gone back to planning menus for the week…’ (FG) • ‘Yesterday I was breathing, when the kids were annoying I was like [sounds of deep breath-in] and I would never have done that.’ (FG) • ‘We had two new members in [a support group] and we had to introduce ourselves and I actually, for the first time, spoke about myself as well as my children…’ (FG) • ‘… [my son] is picking up on the fact that on a Monday [after the session] I feel very good when I come back… and he’s noticing it, so it’s having an impact on how they see us as well…. I felt rot bottom coming to this course and I feel as a person my confidence has grown over the past few weeks. And kids are noticing it… I think each of us from week one we’ve grown.’ (FG)
Impact and changes at 2-month follow-up	• ‘I have actively seen my GP for a carer’s assessment; sought help with dealing with social service through Health Watch’ (FF) • ‘Looking after myself more’ (FF) • ‘I use my awareness of CLANGERS every day’ (FF) • ‘Increased resilience even when things are tough’ (FF)
Most valued elements of the programme	
Learning about CLANGERS	• ‘Gives me much more appreciation of CLANGERS and how using them can improve health and wellbeing.’ (FF) • ‘Opening your eyes to how they [CLANGERS] can improve life.’ (FF) • ‘Having the CLANGERS idea.’ (FF) • ‘Taking note of elements of wellbeing and thinking about what would benefit me and my family’ (FF)
Developing awareness, confidence and learning to focus on own health & wellbeing	• ‘A lot of it is common sense but it is recognising these things and raising the awareness, and thinking about it more.’ (FG) • Participants reported that keeping the ‘CLANGERS diary’, or reviewing in the session which CLANGERS they did, helped them raise awareness and confidence; ‘Even the table to write down, trying that for a couple of weeks, write down what you’ve done and it does give you a boost of confidence because you do quite a lot, and a lot of active stuff, or I wasn’t doing so much of that so what could I do to fulfil that.’ (FG) • ‘You gain confidence with doing the course cause it emphasises that actually we are the most important people. If we don’t look after ourselves as parent carers, it can have a negative effect on the kids.’ (FG) • ‘Learning and putting emphasis on us instead of kids…’ (FG) • ‘Learning how to put yourself first.’ (FF) • The ‘CLANGERS diary’ prompted one participant to take time for herself and keep a journal; ‘It also makes doing that form each week into some sort of journal as well. So you get used to spending that 10, 20 min journaling… It’s time for you, allocated 15 min to fill in that sheet.’ (FG)
Meeting other parent carers, sharing and peer support in non-judgmental, empathetic setting	• ‘Meeting others who understand the situation instead of judgement’ (FF) • ‘I think the main benefit is actually meeting other parent carers.’ (FG) • ‘…in the group we are not alone with this, we all do this.’ (FG) • ‘Being able to gain thoughts and ideas from other parent carers who cope with similar or harder situations’ (FF) • ‘They’ve been through it and they can help you without judging.’ (FG) • ‘Ability to share experiences with others’ (FF) • ‘Peer support very helpful’ (FF) • ‘Peer support, working on being a healthy parent, confidence building, awareness.’ (FF) • ‘Being with others that are in the same boat.’ (FF) • ‘And also in the group we are not alone with this, we all do this.’ (FG)
Positive group interaction	• ‘That there was laughter’ (FF) • ‘Being able to talk openly’ (FF) • ‘Being able to participate and not just being talked to’ (FF) • ‘The active participation of group members - sharing ideas, solutions, being self-reflective’ (FF)
Practical group activities	• Participants liked practical, ‘crafty’ activities, such as making a paper box, the compliment flower, colouring, which were perceived positively and as small achievements in the sessions (FF & FG) • They also liked a group walk (FF & FG)
Ambivalent elements and main suggestions for improvements	
Goal setting	• Some participants felt that they set unrealistic goals; not achieving their goals had a negative effect; e.g.: ‘I found with my goals, looking back, they were probably unrealistic. So although I was doing the CLANGERS every week, I wasn’t achieving my goal. So then I felt guilty and disheartened.’ (FG) • Some felt that setting and reviewing goals was helpful as it raised self-awareness and helped identify barriers; e.g.: ‘Just becoming more aware. It brings up things like “why haven’t I done that?” or “have I been doing that?” which has been really good. And for me just having that awareness is a good starting point because then long-term it will benefit me.’ (FG) • Others felt that there should be more focus on thinking about long-term goals; ‘I think it would have been good to think “these are all the things I want to do long-term’ so setting long-term goals, but in that particular week all goes wrong and you don’t even think about or worry about your goal setting, and you come in and you think “oh no, I’ve not done it”. • Overall, participants agreed that there should be more focus at the beginning of the programme on discussing goal setting (e.g. why and how to set goals).
Contact time and time management in the sessions	• Participants reported that they would welcome more sessions (or on-going groups) that would provide more time to discuss issues related to CLANGERS and other issues that they wanted to share in the group and for peer support; ‘It could have done with another week or two. Especially the second week when we did the Connect, that was quite a big issue for some and we could have done a lot more time on it, cause we had to park stuff but we never actually got back to the parked things cause we didn’t have the time to.’ (FG) • They also would like to have more time within the sessions for ice-breaking, goal setting, unstructured group discussions and filling in feedback forms.
Managing group interaction	• Although participants generally found the group positive and enjoyable, they also felt that sometimes the group dynamics were challenging as everyone wanted to talk about their experiences and issues in a limited time; ‘Also, if [the facilitator] would start this side of the room doing feedback and said ‘let’s just have one’, we’d start with one and by the time we got to the other end then we suddenly went onto everything.’ (FG) ‘There are going to be times where everybody is going to feel they have something else to say and they really want to expand on what they’ve said, and having that opportunity – but it’s just when everyone stops? And with some topics perhaps we could’ve done with a little bit more time to allow to just get that off, when we really needed to off load something.’ (FG) • Participants suggested that it might be helpful to better manage how much time people take talking about their own experiences and views, mixing up where people sit and who they work with, and revisiting group ground rules more regularly.
Feedback forms and questionnaires	• Participants felt there should be more time to fill them in. • They preferred feedback forms specific to each of the CLANGERS. • They would like to be able to report any other circumstances (e.g. recent health issues) affecting their health and wellbeing, and longer-term follow up.

Abbreviations used in the table: *FF* participants’ feedback forms, *FG* focus group

The focus group was attended by six out of seven participants. The themes identified, with exemplary quotes, are shown in Table 7. Overall, participants reported very positive experiences of the programme and the group sessions; they found them informative and enjoyable, and as having a generally positive impact on their health and wellbeing. The main benefits reported were developing confidence, realising the importance of taking care of themselves and their own health and wellbeing, becoming aware of CLANGERS, and peer support. The participants especially liked ‘crafty’ and practical activities, such as creating a box or going for a walk. However, some participants reported that they found setting and failing to meet weekly goals disheartening, and suggested more focus on constructive problem solving and learning to set achievable goals earlier in the programme. In relation to programme delivery, the participants valued having peer facilitators who were understanding and supportive. Six weekly sessions was acceptable contact time, but the participants would also welcome more sessions or if the group was on-going. The participants thought that the programme could also be delivered to existing parent carer groups, thus allowing for longer-term contact and support.

### Indicators of intervention impact

At least half of the participants found each of the sessions helpful in improving their health and wellbeing, and a majority were willing to make some changes as a result of attending the sessions and the programme (Table 6). Only in Session 5, which focused on Relax and Sleep, did half of the participants report that they would not make any changes as a result of the session; their comments indicated that this was due to issues affecting their sleep that they needed to address first (e.g. current health problems, anxiety, child’s sleep problems). At the end of the programme five out of six respondents found the programme helpful in improving their health and wellbeing.

At 2-month follow-up participants continued to perceive a positive impact of the programme (mean satisfaction score 4.2 on a scale 1 to 5). Four out of five respondents assessed the programme as very helpful (scored 4 or 5). These four participants reported also making lifestyle changes (e.g. walking and swimming, taking more notice, having a ‘CLANGERS day’). One respondent reported not finding the programme helpful in improving her health and wellbeing (scored 2), not making any changes as a result of the programme (scored 2), and commented that she found the programme negative and that she had already followed some guidelines. Finally, four out of five respondents reported staying in touch with other group members through other support group meetings or social media.

**Fig. 2:**
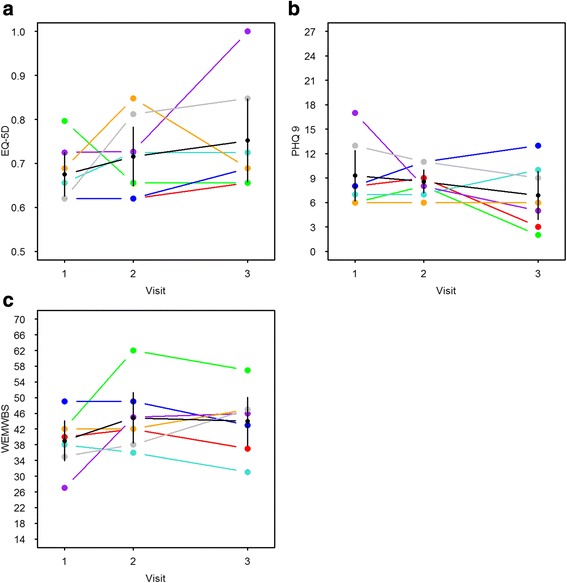
Participants’ scores on the health and wellbeing measures. **a** Changes in participants’ health utility scores (EQ-5D). **b** Changes in participants’ depression symptoms (PHQ-9). **c** Changes in participants’ wellbeing scores (WEMWBS). The (**a**)–(**c**) show changes in individual participant scores (coloured lines), the mean scores for each participant with 95% confidence intervals (black lines) at the three measurement times (baseline, post-intervention and 2-month follow up)

All participants completed the three health and wellbeing questionnaires at each of the three time-points. There was wide variability in individual scores and a trend for change in scores on all questionnaires. The baseline EQ-5D health utility scores indicated poor health with mean baseline score of 0.68 (s.d. 0.067) (Fig. 2a). The baseline PHQ-9 depression scores showed some indications of moderate depressive symptoms (Fig. 2b) with mean baseline score of 9.3 (s.d. 4.2). Similarly, the WEMWBS wellbeing scores suggested low wellbeing scores with a mean baseline score of 39.0 (s.d. 6.8) (Fig. 2c). Overall, at the end of the programme there was a marginal increase in health utility scores (0.72, s.d. 0.09), decrease in depression scores (8.6, s.d. 1.9), and an increase in wellbeing scores (44.9, s.d. 8.7). These patterns were sustained at 2 months after the programme finished (EQ-5D 0.75, s.d. 0.13; PHQ-9 6.9., s.d. 44.0; WEMBS 44.0, s.d. 8.3).

### Incorporating feedback and refining the intervention

The feedback and suggestions for programme improvements from participants and facilitators, and the lessons learned from this feasibility study were summarised and, where possible, incorporated in the revised intervention design. In particular, the Facilitator Manual was revised to include suggestions for delivering the programme and facilitating the groups, and some group activities were added or removed. The main participants’ suggestions for intervention improvements are listed in Table 7. Issues relevant to study design and intervention implementation and adoption are being incorporated in the currently on-going Step 6 ‘Planning intervention adoption, implementation and evaluation’.

## Discussion

The HPC programme was developed using a systematic, user-led approach to promote the health and wellbeing of parent carers. We followed the Intervention Mapping process for developing a health promotion intervention and co-created it with parent carers as the intended end users. The HPC programme was found to be feasible to deliver, acceptable to parent carers and peer facilitators, and has potential to improve health and wellbeing of parent carers. Thus, we consider it a pragmatic first proof of principle of the programme feasibility and acceptability. One crucial aspect of the HPC programme, acknowledged by parent carers involved in the programme development and in the feasibility study, is giving oneself ‘permission’ to focus on your own health and wellbeing.

The parent carers who participated in the study had indications of poor health and wellbeing with low health utility scores, similar to samples of people with chronic conditions [45]; baseline PHQ-9 scores suggesting a moderate risk of depression; and, for six of the seven participants, wellbeing scores considerably lower than population norms for the WEMWBS in the Health Survey for England data from 2011 [46]. These results confirm the findings of the needs assessment and indicate that it is possible to recruit the target population.

Although we had concerns about being able to reach participants from diverse socio-economic circumstances, our participants came from a range of backgrounds, including some living in relatively more deprived areas. However, one aspect of the study that did not meet our criteria for ‘success’ was the rate of recruitment. We began recruiting in December, as soon as ethics approval was confirmed. Although a difficult time to get parents’ attention, we hoped that recruiting over Christmas and beginning the sessions in a New Year might be advantageous as it is a time when many people formulate intentions to improve their health and/or wellbeing. However, recruitment was lower than expected. This might have been, in part, due to parent carers’ being busy with family responsibilities over Christmas holidays. We also learned that some of the parent carer organisations, through which we hoped to advertise, had ceased sending out emails to their parent carer mailing lists. Thus, recruitment for future studies should work more closely with stakeholder organisations that can help with reaching and recruiting parent carers and/or recruiting existing parent carer groups.

The time needed to attend 6 weekly, half-day sessions was not a barrier to participating in the programme. Indeed, participants stated in the focus group that they would welcome a longer programme. Although we offered a one-off, introductory group session, interest in this session was so low we decided not to proceed with it.

Although the programme focuses on promoting health and wellbeing on an individual level (i.e. individual-level psychological and behaviour change), we acknowledge the importance of other factors on inter-personal, community and societal levels that affect parent carers’ health and wellbeing. For example, societal factors, such as access to services or negative public attitudes towards disability can have a huge impact on parent carers’ wellbeing. Whilst the HPC programme may help with handling the consequences of these factors through increased empowerment and resilience, the programme does not aim to provide guidance on the practical strategies for obtaining rights or navigating the healthcare system. The programme included signposting to sources of advice in the UK, such as Cerebra’s legal toolkit and advice [47] and Council for Disabled Children’s ‘Expert Parent Programme’ [20].

This study has significant limitations. The sample was small, self-selecting and homogeneous in terms of gender and ethnicity. Thus, the sample is not representative of the population. The lack of ethnic diversity in South West England, where this study was conducted, limits the generalisation of our findings to different cultures and contexts. Compared to other areas of United Kingdom, the South West has the highest proportion of people declaring themselves ‘white British’. Ethnic and cultural factors may well influence the uptake and implementation of the intervention and merits further research.

The feasibility study (part of intervention development) did not include a comparison group so that no clear inferences can be made regarding effectiveness or generalisability. Offering group sessions only during school-times might have precluded recruitment of parent carers unavailable at these times (e.g. working parents). The group was delivered by two facilitators experienced in delivering training and support groups to parent carers, and who had been involved in the programme development; thus, they were skilled and knowledgeable about the ethos and content of the programme. Finally, we did not specifically assess whether participants engaged with CLANGERS or changed their behaviours in result of the sessions/programme (although we asked them for intentions and examples in the session feedback forms and in the focus group). Future research should address these issues, for example, by adding a comparison group, offering group sessions on different days, times and places, assessing the fidelity of delivery and participants’ perceptions when the programme is delivered by different facilitators, and assessing changes in behaviours and other intermediary factors hypothesised to affect health and wellbeing.

The HPC programme was developed systematically using an Intervention Mapping approach [14]. We found this methodology challenging as it requires considerable resources to complete tasks and relies on an existing evidence base specific to the population and context. As we had limited time and resources, found little high-quality research focusing on parent carers, and wanted to include psychological as well as behavioural outcomes, we had to adapt the methods. For example, we were unable to conduct a full-scale systematic review of health promotion interventions for parent carers (although we identified some helpful reviews) or to explore systematically (e.g. through a qualitative study) parent carers’ views on health promotion. However, as we worked closely with parent carers and stakeholders throughout the study, we believe our methodology was robust.

## Conclusions

The Healthy Parent Carers programme was co-created and tested with parent carers and appears to be a promising health promotion intervention for parent carers. This study has led to refinement of the intervention and the next stage of testing is being planned. The programme purposefully promotes relatively simple messages, and small, achievable steps, which have been tailored to the context of parent carers’ lives. Actively promoting health and wellbeing is critical if we were to ensure better quality of life of parent carers and their children and families.

## Abbreviations

6SQuID: 6 Steps in Quality Intervention Development
BCTs: Behaviour Change Techniques
CLANGERS: Connecting with people, continuing Learning, being Active, taking Notice, Giving, Eating well, Relaxing, and maintaining Sleep hygiene
EQ-5D: EuroQol 5 Dimensions Questionnaire
HPC: Healthy Parent Carers
PHQ-9: Patient Health Questionnaire-9
s.d.: standard deviation
SEND: Special Educational Needs and Disability
TIDieR: Template for Intervention Description and Replication
WEMWBS: Warwick-Edinburgh Well-Being Scale
